# Supplementary material for: Interlayer donor-acceptor pair excitons in MoSe2/WSe2 moiré heterobilayer
Source: Nat Commun. 2023 Sep 18;14:5766. doi: 10.1038/s41467-023-41330-6 (PMC10507070; doi:10.1038/s41467-023-41330-6)
Supplement: Supplementary file 1 — Supplementary Information [file 41467_2023_41330_MOESM1_ESM.pdf]

Supplementary information:

Interlayer donor-acceptor pair excitons in $MoSe_2/WSe_2$ moiré heterobilayer

Section 1. PL spectrum peak analysis

This section discusses how we estimate the band-edge IX and DAP IX peak positions. We first discuss the band-edge IX peak determination. Using the band-edge IX peak energy and considering the DAP IX peak linewidth, we then determine the minimum energy of DAP IX. Finally, we discuss how we use the DAP peak formula to obtain the predicted DAP IX peaks.

For the band-edge IX, considering the coupling between band-edge and DAP IXs, its peak energy (E_{IX}) should be close to the dominant DAP IX peak (i.e., the IX energy with the brightest PL emission). Additionally, due to the non-uniform energy distribution of the donor-acceptor pair (i.e., there are more donor-acceptor pairs with lower energy), E_{IX} should be larger than the dominant DAP IX peak. Hence, we perform the following procedure to obtain E_{IX}. We first perform a single Gaussian fitting on the IX PL spectrum to get the position of the dominant DAP IX peak. Then we fit the PL spectra with multiple Lorentzians (representing the DAP IX) plus a single Gaussian (representing the band-edge IX), imposing that the Gaussian peak energy must be larger than the dominant DAP IX peak. We take the resulting Gaussian peak energy as E_{IX}. We obtain E_{IX} equal to 1.384 eV, 1.387 eV, and 1.378 eV for dot 1, dot 2, and dot 3 in the main text, respectively. We note that this is the neutral band-edge IX. As described in the main text, from the energy dependence of the lifetime, we found another band-edge IX at lower energy, attributed to the trion peak.

To get the minimum energy of DAP IX (E_0), we consider both the DAP IX linewidth and the band-edge IX. The distance between two adjacent separable Lorentzian peaks should be larger than 60% of their full-width-at-

half-maximum (FWHM). Using this criterion, we obtain $E_0 \le 1.33\,\mathrm{eV}$. Next, we assume that the difference between E_0 at two different dots should be similar to the difference between the corresponding E_{IX} since, like the E_{IX}, the value of E_0 is also affected by the local strain. We chose the highest E_0 possible for all the dots fulfilling the criteria explained above, obtaining the following E_0: 1.327 eV (dot 1), 1.33 eV (dot 2), and 1.32 (dot 3)).

For determining the peak position of DAP IX, we need to obtain the value of the following parameters beside E_0 (see Eq. (1) in the main text): dielectric constant (ε) and donor-acceptor distance (R_m). We used $\varepsilon = 7.55\varepsilon_0$ based on the $MoSe_2$ and WSe_2 out-of-plane dielectric constant[1]. For R_m, since the lattice constant of $MoSe_2$ is approximately the same as WSe_2, it can be expressed as

$$R_m = R(m_1, m_2) = \sqrt{\delta^2 + a_0^{\,2}\left(m_1^{\,2} + m_2^{\,2} + m_1 m_2 + m_2 + \frac{1}{3}\right)}, \quad \text{(S1)}$$

where m is the shell number (with R_m is larger for larger value of m), m_1 and m_2 are integers, $\delta = 0.7\,\mathrm{nm}$ is the interlayer distance as reported in the literature[2,3], and $a_0 = 0.33\,\mathrm{nm}$ is the lattice constant of $MoSe_2$ (WSe_2).

However, Eq. (S1) is only correct if there is no strain variation in the sample, which is not the case for the moiré heterobilayer. For $MoSe_2/WSe_2$ heterobilayer, the strain variation (σ_S) due to moiré superlattice can reach ±1.5%[4], resulting in the uncertainty in R_m and, thus, in calculated DAP IX peak energy. Based on Eq. (1) in the main text, the uncertainty in DAP IX peak energy due to strain variation can be expressed as

$$\begin{aligned}\sigma_E &= \left|E(R_m) - E(R_m + \sigma_R)\right| \\ &= \left(E - E_0\right)\left|\frac{(\sigma_R/R_m)}{1 + (\sigma_R/R_m)}\right| \\ &= \left(E - E_0\right)\left|\sigma_S/(1+\sigma_S)\right| \approx \left(E - E_0\right)\left|\sigma_S\right|, \end{aligned} \quad \text{(S2)}$$

where $E = E(R_m)$ is the DAP IX peak energy calculated using Eq. (1) and (S1), and $\sigma_R = R_m \sigma_S$ is the uncertainty in the value of R_m. From the experimental results, we know that $(E - E_0) \geq 20$ meV. Hence, using $\sigma_S = 1\%$, we obtain $\sigma_E \geq 0.2$ meV. Considering this σ_E, the predicted DAP IX peaks (red lines in Fig. 2(a-c) in the main text) are taken to be the observed peaks that are within ±0.2 meV of the calculated DAP IX peaks (using Eq. (1) and (S1)). Similarly, an observed peak matches the DAP IX peaks if its energy is within ±0.2 meV from the calculated DAP IX peaks.

Section 2. DAP IX energy-lifetime correlation

The fast (k_1) and slow (k_2) decay rates solution for the coupled DAP-band edge IX model (inset of Fig. 3d in the main text) can be expressed as

$$k_1 = \left(\left(k_{DAP} + k_{IX} + 2D \right) + \sqrt{\left(k_{DAP} - k_{IX} \right)^2 + 4D^2} \right) \Big/ 2, \quad \text{(S3)}$$

$$k_2 = \left(\left(k_{DAP} + k_{IX} + 2D \right) - \sqrt{\left(k_{DAP} - k_{IX} \right)^2 + 4D^2} \right) \Big/ 2, \quad \text{(S4)}$$

where $k_{DAP} = k_{nr} + k_{r0} \exp\left(-\dfrac{2\alpha}{R_B (E - E_0)} \right)$ is the DAP IX total decay rate with k_{nr} is the DAP IX nonradiative decay rate, k_{r0} is the DAP IX maximum radiative decay rate, R_B is the largest Bohr radius between the donor and acceptor, E is the DAP IX energy, E_0 is the minimum DAP IX energy, and $\alpha = \dfrac{e^2}{4\pi\varepsilon}$ with e is the electron charge and ε is the effective permittivity, k_{IX} is the band-edge IX decay rate, and D is the coupling rate between band-edge and DAP IX. While k_{nr} and k_{IX} depends on the DAP IX energy (see Section S3), it can be assumed to be constant at low power (as in the case of Fig. 3 in the main text).

The fast and slow decay rates are obtained using Eqs. (S3) and (S4). The maximum radiative decay rate of DAP IX (k_{r0}) is set to be the same as the maximum radiative decay rate of exciton in TMD ~ 250 GHz[5]. The energy

dependence of D and R_B can be expressed as

$$D = \sum_i D_{0(i)} \frac{w_{D(i)}^2}{w_{D(i)}^2 + 4\left(E - E_{IX(i)}\right)^2}, \tag{S5}$$

$$R_B = \sqrt{R_{B0}^2 + \sum_i \Delta_{R(i)}^2 \frac{w_{R(i)}^2}{w_{R(i)}^2 + 4\left(E - E_{IX(i)}\right)^2}}, \tag{S6}$$

where E_{IX} is the band-edge IX central energy and the index i is the band-edge IX index. The values of w_D and w_R (ranging from 2 to 16 meV) are proportional to the band-edge IX linewidth. When there is no coupling, the Bohr radius is R_{B0} ~0.5 nm, comparable to the calculated Bohr radius for some impurity states in monolayer TMD[6]. At maximum coupling, the Bohr radius is increased to ~0.9 nm, which is reasonable given that the radius of the electron (hole) wavefunction in a band-edge IX is ~ 2.4 nm[7].

Section 3. Power dependence of DAP IX lifetime

To capture the power dependence, the following power saturation relations were used:

$$k_{nr}^{DAP(IX)} = k_{nr\text{-}max}^{DAP(IX)} \frac{P}{P + P_{sat}^{DAP(IX)}}, \tag{S7}$$

$$\Delta E_{IX(i)} = \Delta E_{max(i)} \frac{P}{P + P_{sat}^{IX(i)}}, \tag{S8}$$

where $k_{nr}^{DAP(IX)}$ is the nonradiative rate of the DAP (band-edge) IX, $k_{nr\text{-}max}^{DAP(IX)}$ is the maximum nonradiative rate of the DAP (band-edge) IX, $P_{sat}^{DAP(IX)}$ is the saturation power for the DAP (band-edge) IX nonradiative decay, $\Delta E_{max(i)}$ is the (maximum) band-edge IX energy shift, $P_{sat}^{IX(i)}$ is the saturation power for the band-edge IX energy shift, and the index i is the band-edge IX index.

The DAP IX nonradiative recombination is mainly attributed to the Auger process. The power dependence is due to excitation-induced doping. The higher-energy DAP IX has a higher Auger coefficient[8], resulting in the

energy-dependent $k_{\text{nr-max}}^{\text{DAP}}$. Both the Auger process and exciton-exciton annihilation contribute to the nonradiative rate of band-edge IX. Considering that the band-edge exciton and trion can have different nonradiative rates, the value of $k_{\text{nr-max}}^{\text{IX}}$ depends on whether the DAP IX is closer to the band-edge exciton or trion. The energy shift of band-edge IX is mainly attributed to the dipole-dipole interaction.

Considering that different processes involved can have different saturation power, we cannot use one value of saturation power for all processes. In the fitting of dot 3 data (Fig. 4 in the main text and Fig. S11), the values of saturation power used are $P_{\text{sat}}^{\text{DAP}} = 12\ \mu\text{W}$, $P_{\text{sat}}^{\text{IX}} = 28\ \mu\text{W}$, $P_{\text{sat}}^{\text{IX(1)}} = 6\ \mu\text{W}$ for exciton, and $P_{\text{sat}}^{\text{IX(2)}} = 18\ \mu\text{W}$ for trion. The maximum energy shift is $\Delta E_{\max(i)} \approx 8\ \text{meV}$ for both exciton and trion.

Section 4. Role of the moiré superlattice size

To study the effect of moiré superlattice size, we compare the PL spectra taken from samples with different twist angles: near 60° (i.e., the sample in the main text), 0°, and 20(40)° twisted samples. The difference in twist angle results in different moiré superlattice sizes and potential strengths. We found that the number of peaks and the peak linewidth in our PL spectrum depends on the twist angles and, consequently, on moiré superlattice size (Fig. S14). In particular, the 20(40)° twisted sample shows only broad peaks, while the other two samples show multiple sharp peaks. This phenomenon can be well-explained using the DAP IX framework. In particular, due to the moiré potential barrier, the donor and acceptor should be localized within the same moiré cell. Unlike in the other two samples, the 20(40)° twisted sample has a smaller moiré superlattice constant, resulting in less probability of DAP IX formation.

Additionally, we can estimate the maximum donor-acceptor distance of the near 0° twisted sample following the same procedure done for the near 60° twisted sample. In particular, we first find the E_0 by considering that

the distance between two adjacent separable Lorentzian peaks should be larger than 60% of their full-width-at-half-maximum (FWHM ~ 1 meV for the near 0° twisted sample). Using this criterion, we obtain $E_0 \sim 1.303$ eV for the near 0° twisted sample. By using this E_0 and the minimum energy of the detected DAP IX peak (~ 1.327 eV) in the main text Eq. (1), we obtain a maximum donor-acceptor distance of ~8 nm. This value is similar to the near 60° twisted sample case, which is expected given that the moiré superlattice constants in these two cases are similar. Our results on the twist angle dependence show that the moiré superlattice size is essential in the formation of dense and narrow peaks.

Section 5. Defect characterization and donor-acceptor pair nature

There are two possible origins of the localized donor and acceptor energy levels:

1. Impurity/vacancy

   The impurity/vacancy can result in localized energy levels in the band gap.

2. Moiré- and strain-induced localization

   Due to moiré superlattice and external strain, the electron/hole experience can be localized in a single moiré site. Moreover, there can also be lattice reconstruction which can enhance the localization[9]. This localization creates energy levels near the conduction (valence) band, which can also act as donor/acceptor energy levels.

To identify which kind of donor/acceptor sources result in localized DAP IX with an energy of around 1.3 to 1.4 eV, we first characterize the defect in our sample using transmission electron microscopy (TEM). The TEM measurement result of suspended WSe_2 and $MoSe_2$ monolayers are shown in Fig. S15a and S15b, respectively. The possible impurities and vacancies, together with their approximated density, are shown in Fig. S15c. The combined density of V_{Se} and $V_{Se}V_{Se}$ in $MoSe_2$ is ~7 times the expected density of the moiré superlattice (~2×10^{11} cm^{-2} for twist angle of ~1°), while the combined density of V_W and Se_W is comparable to moiré superlattice one.

We then analyze the energy levels corresponding to these defects. The band structure of these defects based on DFT calculation with HSE06 functional[10,11] are shown in Fig. S16. The alignment between $MoSe_2$ and WSe_2 band is obtained by considering band-edge IX energy of 1.4 eV and IX binding energy of 0.32 eV[12]. Considering the high density of defects in $MoSe_2$, it is reasonable to assign the donor part of DAP IX to the defects in $MoSe_2$. From the band energy diagram (Fig. S16), the energy levels of the V_{Se} and $V_{Se}V_{Se}$ defects in $MoSe_2$ are located around 1.3 to 1.36 eV above the VBM, comparable to the value of E_0 (i.e., the minimum DAP IX energy) in our case. This value of E_0 indicates that the acceptor level should be close to the VBM. Such acceptor level can be the moiré/strain-localized energy level near the WSe_2 valence band.

Section 6. Role of the nanopillar-induced strain

We consider four different mechanisms the nanopillar-induced strain can affect the PL intensity, i.e., the strain and strain gradient can

1. create new emitters,
2. modify the moiré superlattice structure,
3. modify the coupling strength between the band-edge and DAP IX, and
4. modify the total IX population inside the excitation beam through the funnelling effect.

We conduct experiments and theoretical analysis to determine which effect above is the most dominant. Firstly, to check if the PL emission is due to the creation of new emitters by strain, we compare the PL spectrum on nanopillar, wrinkle, and flat areas (Fig. S6). Like in the nanopillar case, we found that PL signals from other areas also consist of dense and narrow peaks, albeit with weaker PL intensity. Such observation shows that the primary role of the strain is to enhance the PL intensity instead of creating new emitters. This strain enhancement of the PL intensity can also explain why the PL intensity from the wrinkle area is higher than that from the flat

area. Compared to the Raman spectrum at the flat area, the Raman spectrum at the wrinkle area shows an apparent redshift of WSe_2 A'+E' phonon mode[13] (Fig. S17), indicating a larger strain and, thus, higher PL intensity.

Next, we check if the strain-induced modification moiré superlattice structure is responsible for the PL intensity enhancement. To do this, we first conducted TEM characterization on the flat and nanopillar area to probe the structure modification due to nanopillar. For this purpose, we fabricate a new sample where the heterostructure sample sits on suspended nanopillars on hBN layer support (see Fig. S18), allowing the detection of electron transmission through the sample. The TEM and selected area electron diffraction (SAED) images from this heterostructure sample on nanopillar (diameter: 100-200 nm, height: ~130 nm) and flat areas are shown in Fig. S19 (upper and lower images correspond to areas around two different nanopillars). These images show that the twist angle in the nanopillar and flat areas can be quite different, while the monolayer lattice constant does not change much (see Fig. S20 for the linecut of the SAED images extracted from the upper images of Fig. S19). However, the nanopillar-induced strain can decrease or increase the moiré superlattice size with no apparent trend. Hence, we cannot conclude that this size change is responsible for the PL enhancement. Instead, we conclude that the role of strain is mainly the effect no. 3 and no. 4 in the list above, i.e., PL intensity enhancement through two mechanisms: strain-enhanced coupling between the band-edge and DAP IX, and strain gradient-induced funnelling. More quantitative discussion on the PL intensity enhancement by nanopillar-induced strain is given in Section 7.

Section 7. PL intensity enhancement by nanopillar-induced strain

To study the role of the nanopillar-induced strain on the PL intensity enhancement more quantitatively, we compared the PL intensity from the nanopillars shown in Fig. S5. All nanopillars have the same height of ~130 nm, with the distance between nanopillars being 4 μm. The pillar diameter, theoretical strain, the PL intensity

(obtained from the intensity map in Fig. S5), and the coupling strength for each nanopillar are compiled in Table S1.

The theoretical strain is obtained from the numerical simulation of a membrane deposited on a SiO_2 nanopillar substrate. Considering there is a 10-nm thick hBN between the thin TMD heterostructure and the SiO_2 substrate, the membrane is modelled as 10 nm hBN membrane (Young's modulus, $Y = 0.8\,\text{TPa}$; Poisson's ratio, $\nu = 0.2$; mass density, $\rho = 2100\,\text{kg.m}^{-3}$) in the COMSOL plate model. The van der Walls interaction between the membrane and the substrate is modelled using Lennard-Jones (LJ 6-12) potential[14]

$$V(r) = \Gamma_\text{a}\left(\left(\frac{d_\text{e}}{r}\right)^{12} - \left(\frac{d_\text{e}}{r}\right)^{6}\right), \tag{S9}$$

where r is the distance between the substrate surface and a point on the membrane, $\Gamma_\text{a} = 0.2\,\text{J.m}^{-2}$ is the hBN-SiO2 interfacial adhesive energy[15], and $d_\text{e} = 0.6\,\text{nm}$ is the equilibrium interlayer distance without strain. The nanopillar substrate exerts van der Waals force on the membrane in the direction normal to the substrate surface (see Fig. S21a). The solution to the plate model is obtained using COMSOL. The two-dimensional simulated strain profile for pillar diameter, $d = 200\,\text{nm}$, is shown in Fig. S21b, showing an expected circular rotational symmetry. The plot of strain vs the distance from the centre of the pillar for various pillar diameters is shown in Fig. S21c.

Considering the coupling between DAP and band-edge IX, the collected DAP IX PL count rate can be expressed as

$$I_\text{PL} = C_0 \eta_\text{D} d_\text{int}^2 N_\text{IX}, \tag{S10}$$

where N_IX is the IX population within the excitation beam diameter, d_int is the diameter of the effective interaction area, η_D is the coupling efficiency between DAP and band-edge IX, and $C_0 = \pi \eta_\text{coll} k_\text{r} n_\text{DAP}/4$ is taken

to be constant where n_{DAP} is the number of donor-acceptor pair per area, k_{r} is the average radiative decay rate of DAP IX, and η_{coll} is the collection efficiency. The value of d_{int} and N_{IX} depends on the band-edge IX transport. For excitation at the nanopillar, the IX transport is mainly affected by the funnelling, which depends on the strain gradient. The strain gradient is negligible within the nanopillar area, and it does not have a strong dependence on the pillar diameter outside the nanopillar area (see Fig. S21c). Hence, for excitation on the nanopillar, the band-edge IX population will be concentrated in the nanopillar area, i.e., $d_{\mathrm{int}} \approx d$, and N_{IX} does not depend on the pillar diameter. The coupling strength for the excitation on nanopillar can then be expressed as

$$\eta_{\mathrm{D}} = \frac{I_{\mathrm{PL}}}{C_0 N_{\mathrm{IX}} d_{\mathrm{int}}^2} \propto \eta_D^{\mathrm{exp}} = \frac{I_{\mathrm{PL}}}{d^2}, \tag{S11}$$

where η_D^{exp} is the experimentally derived coupling strength metric. The values of η_D^{exp} for the nanopillars calculated using Eq. (S11) are included as the coupling strength in Table S1.

For excitation on the flat area, the band edge IX transport is mainly determined by the IX-IX repulsion and disorder. In the absence of disorder (e.g., nanobubble), the effective interaction area will be the same as the excitation area. However, the disorder will reduce this value. On the other hand, the IX-IX repulsion spreads the band-edge IX population, resulting in a smaller N_{IX} than the one in the case of nanopillar. Considering these two factors, the coupling strength for the excitation on the flat area can be expressed as

$$\eta_D^{\mathrm{exp}} > \frac{I_{\mathrm{PL}}}{d_{\mathrm{beam}}^2}, \tag{S12}$$

where $d_{\mathrm{beam}} \sim 500\,\mathrm{nm}$ is the beam diameter. The lower boundary of η_D^{exp} for the flat area calculated using Eq. (S12) is included as the coupling strength in Table S1.

Figure S21d shows the plot of η_D^{exp} as a function of the strain (σ) on the pillar. We also included the lower

boundary of $\eta_D^{\exp}$ in flat area (i.e., zero strain) in this figure. We observed that, generally, a higher strain results in higher coupling strength. This result is agreeable with the coupling mechanism shown in Fig. 1b. More specifically, this coupling mechanism will have a Lorentzian dependence on the energy difference between the band-edge and DAP IX. The band edge IX energy is linearly redshifted by strain[16]. On the other hand, the defect-related energy levels are less sensitive to strain and only experience redshift at high strain[17]. As a result, the strain dependence of the coupling strength should have a Lorentzian shape at low strain and saturate at high strain. In Fig. S21d, we have included the Lorentzian fitting of the experimental data, showing a good fit.

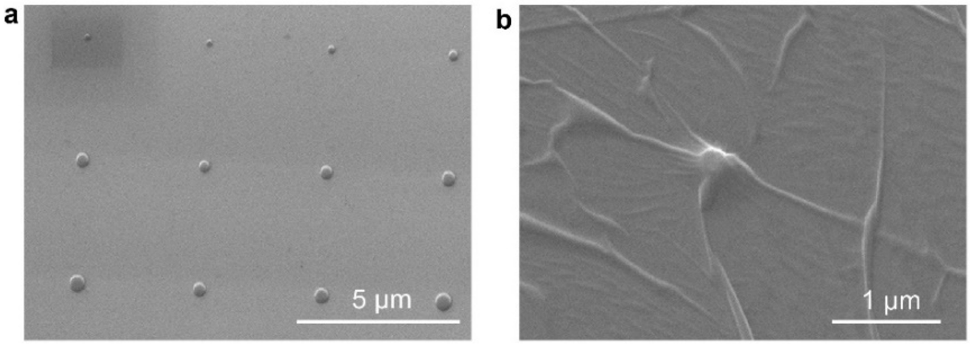

Figure S1 | SEM image of the sample. a, b SEM image of the sample showing the nanopillar array and the heterostructure laid on top of it. The alignment between the sample area in (a) and the one in Fig. S5 is shown in Fig. S13.

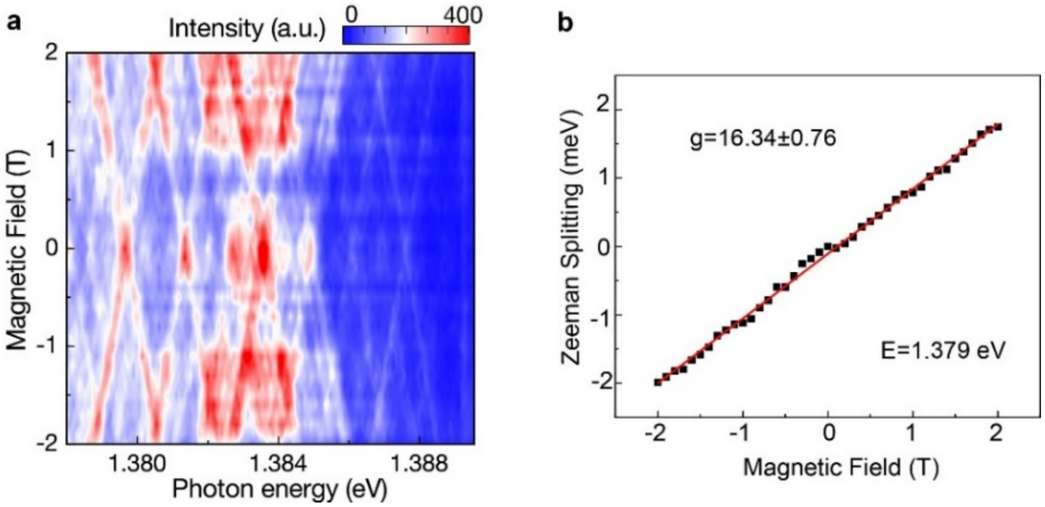

Figure S2 | Magnetic field dependence of LIX PL spectrum. a, LIX PL spectrum as a function of the applied out-of-plane magnetic fields at 4.2 K under 765 nm (1.62 eV) laser excitation. Circular polarization filters were not used in the collection and excitation part. An 850 nm long pass filter is used in the collection arm. **b,** Magnetic field dependence of the Zeeman splitting for one of the LIX peaks (with emission energy of 1.379 eV @ 0 T peak). The linear fitting shows an effective *g*-factors of 16.34±0.76.

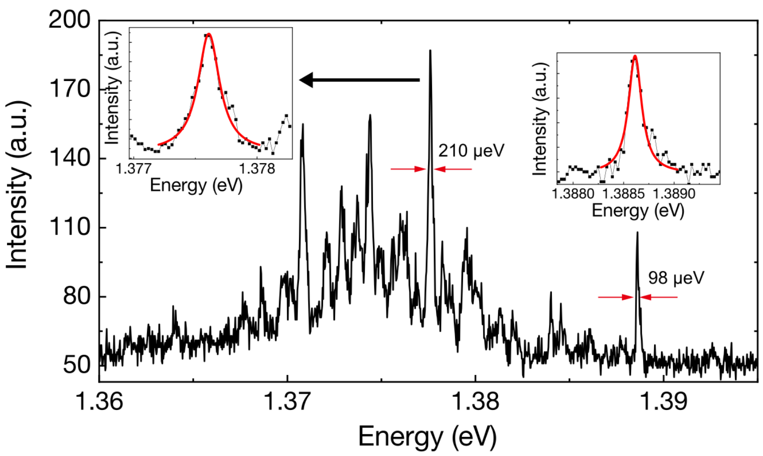

Figure S3 | Typical linewidths of LIX peaks. Lorentzian fittings of LIX peaks show FWHM of 210 μeV (for the 1.377 eV peak) and 98 μeV (for the 1.389 eV peak). The experimental condition is the same as in Fig. S2.

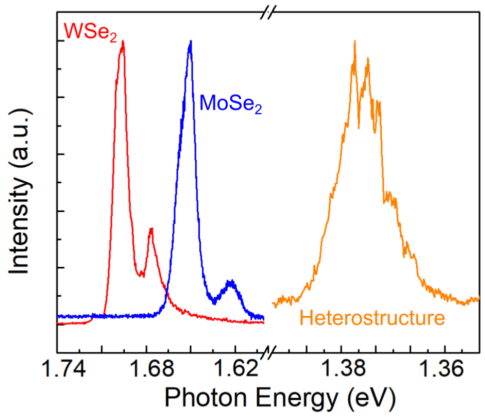

Figure S4 | Typical PL spectrum under a 532 nm laser excitation at flat monolayer $MoSe_2$, flat monolayer WSe_2, and the heterobilayer on nanopillar area.

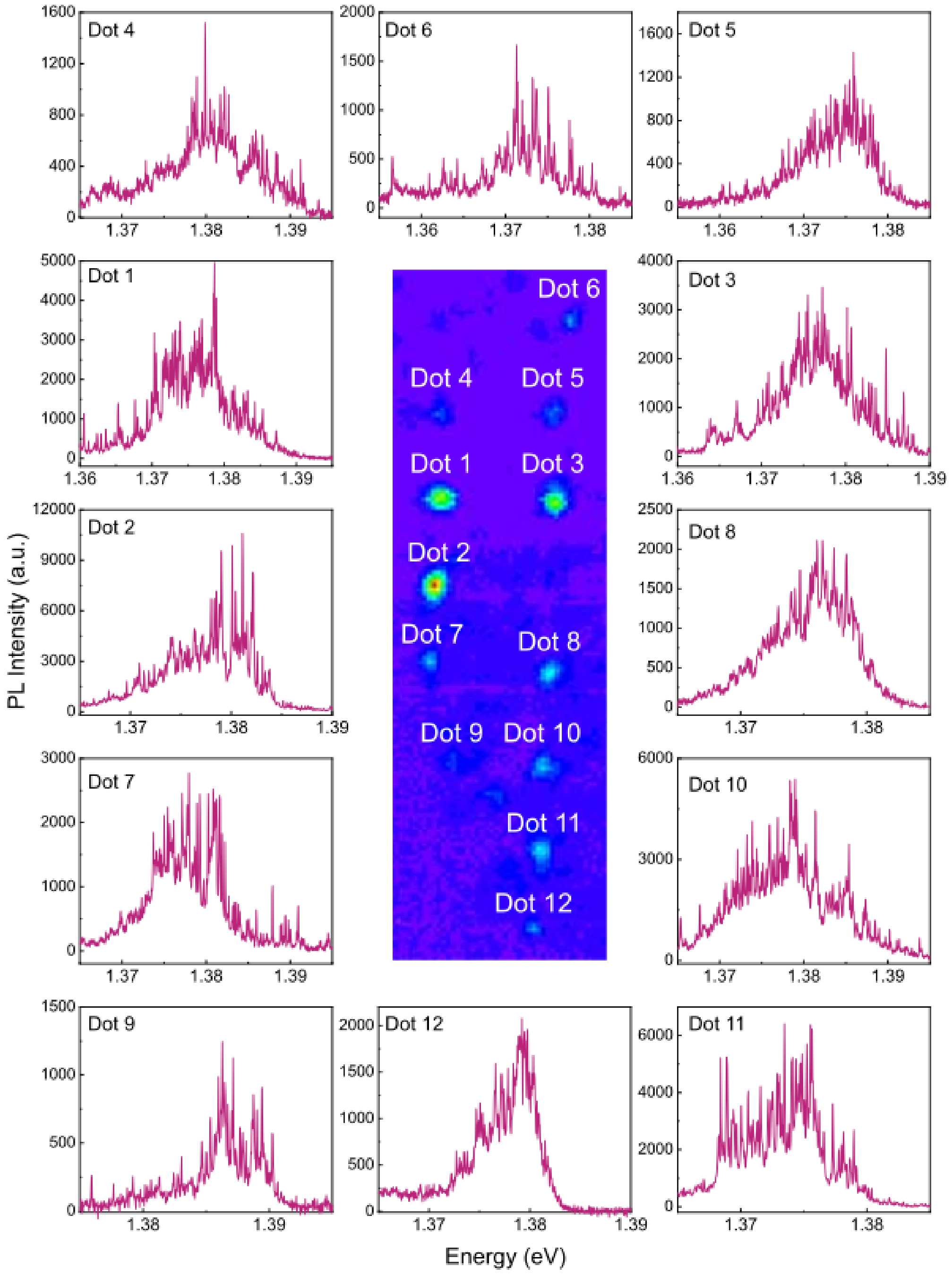

Figure S5 | LIX PL spectra from various nanopillars. Middle: Integrated PL intensity map. The integrated PL intensity map shows a significant intensity enhancement at nanopillars. Around: PL spectra at various excitation locations (labelled as Dot 1 to Dot 12 on the map). The IX PL spectra show multi peaks with the FWHMs ranging from 100 to 800 μeV. The experimental condition is the same as in Fig. S2. The dimensions of the nanopillars can be found in Table S1.

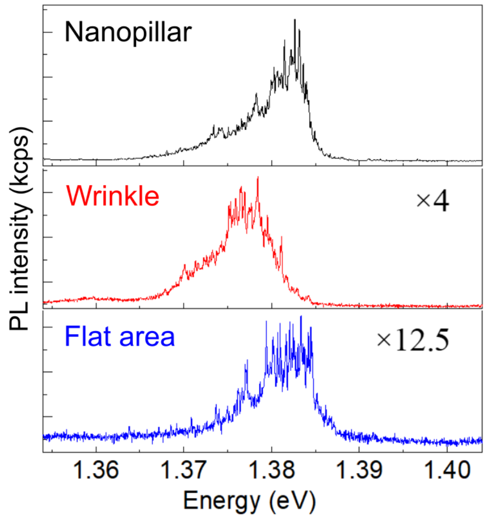

Figure S6 | Comparison between PL spectrum from different areas on the same sample. The excitation power is ~40 nW. Dense and sharp peaks are observed in all spectra. The PL spectra from the wrinkle and flat areas are multiplied by 4 and 12.5 times, respectively. The distance between the major ticks is 250 cps.

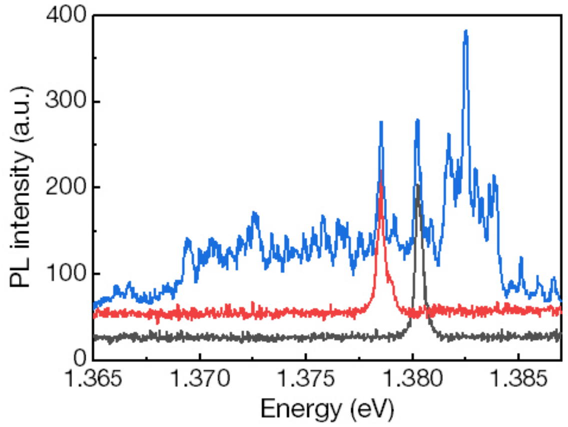

Figure S7 | Grating-filtered LIX PL spectrum. The blue line is the IX PL before the filtering by the grating. Two typical LIX PLs after filtering are shown as red and black lines, showing an FWHM of around 700 μeV.

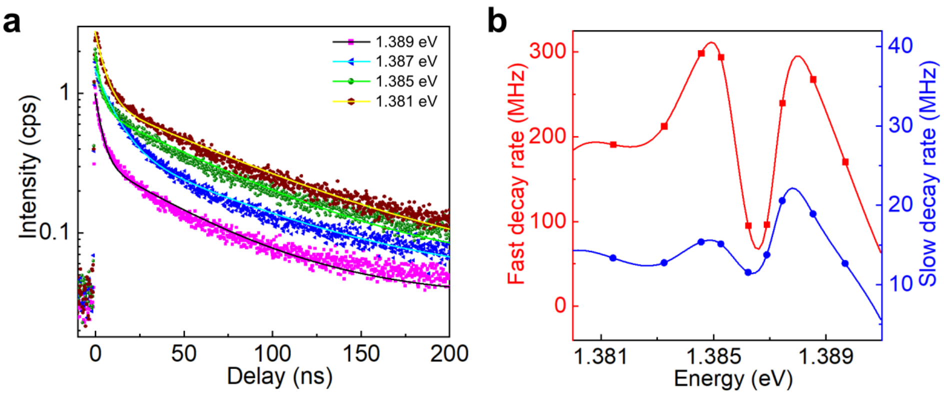

Figure S8 | Energy dependence of the PL dynamics in the flat area. a, Energy- and time-resolved PL of LIX emission from the flat area. Data (shown as symbols) was obtained under 100 nw excitation. The lines are double exponential decay fitting results. The error bars are smaller than the symbol size. **b,** Decay rates vs emission energy. The lines are guides for the eye. Two maxima with energy separation of ~5 meV are detected, corresponding to band-edge neutral exciton and trion.

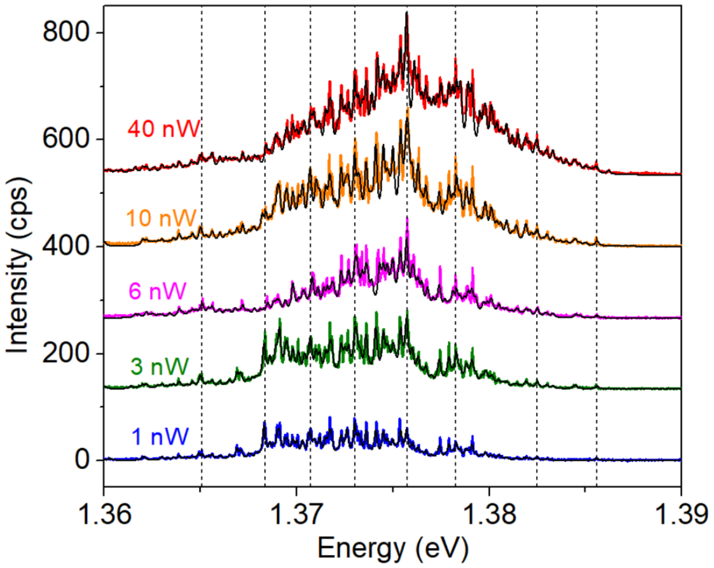

Figure S9 | Lorentzian fitting of IX emission at various powers. Solid black lines are multiple Lorentzian fittings. The dashed lines indicate the tracking of some peaks. There is no clear power-dependent blue shift observed.

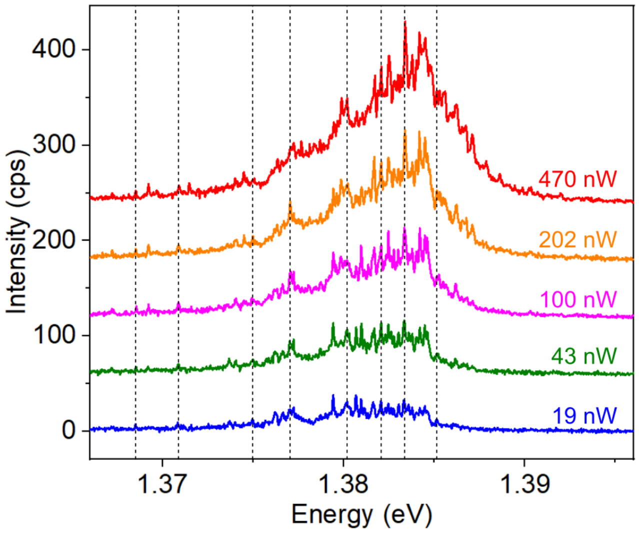

Figure S10 | Power dependence of the PL from the flat area. The dashed lines indicate the tracking of some peaks.

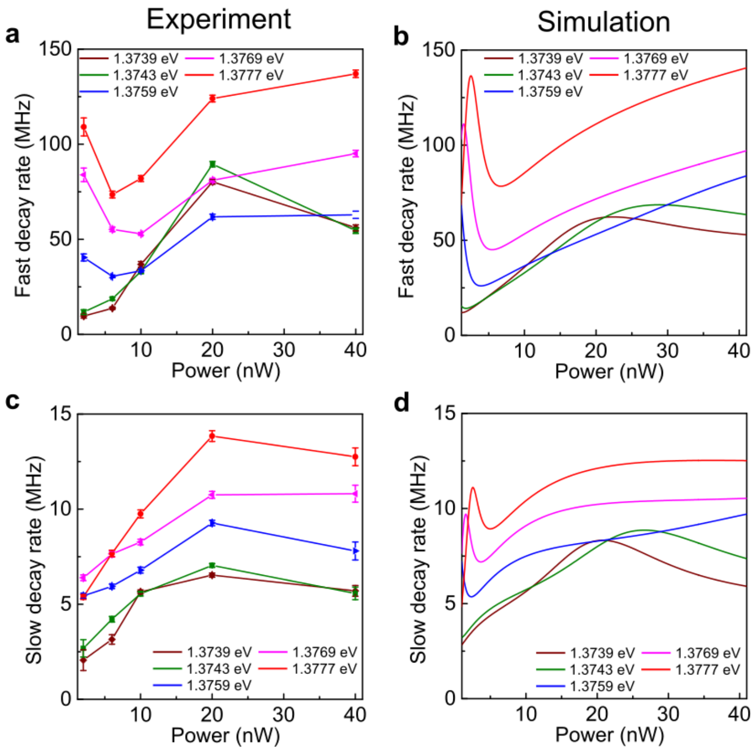

Figure S11 | Power dependence of decay rates. a, Experimental and **b,** simulated fast decay rates vs excitation power. **c,** Experimental and **d,** simulated slow decay rates vs excitation power.

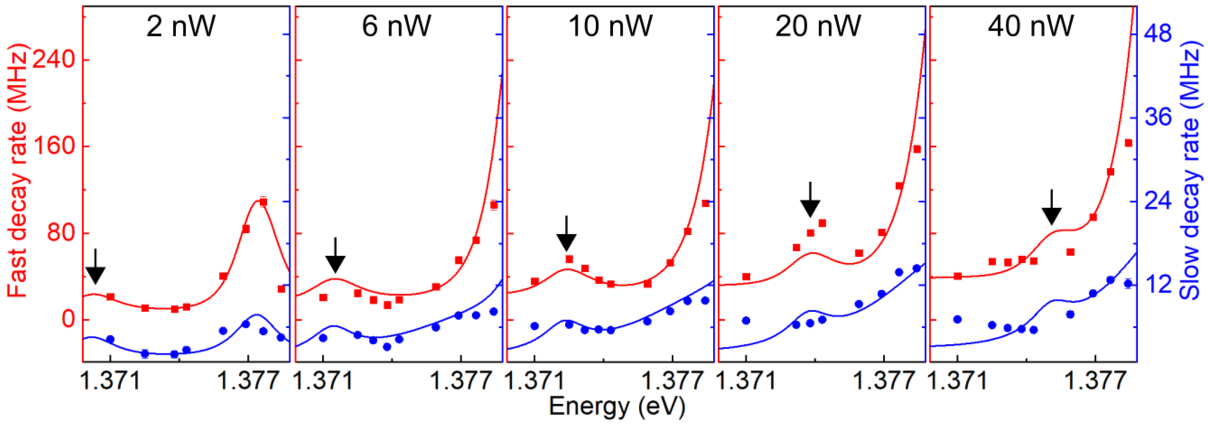

Figure S12 | Power dependence of lifetime-energy correlation of dot 3. The black arrows show the position of the band-edge trion peak. The symbols are measurement results, and the lines are theoretical calculations.

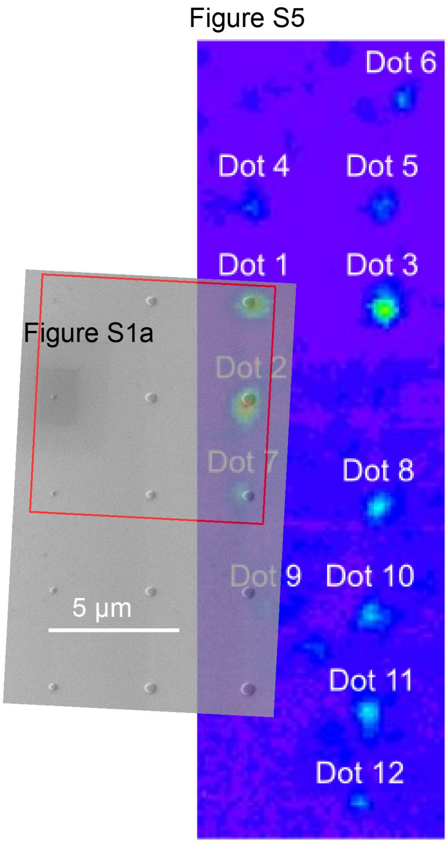

Figure S13 | Alignment between different areas of the sample. The red box indicates the pattern set repeated over the sample during the fabrication.

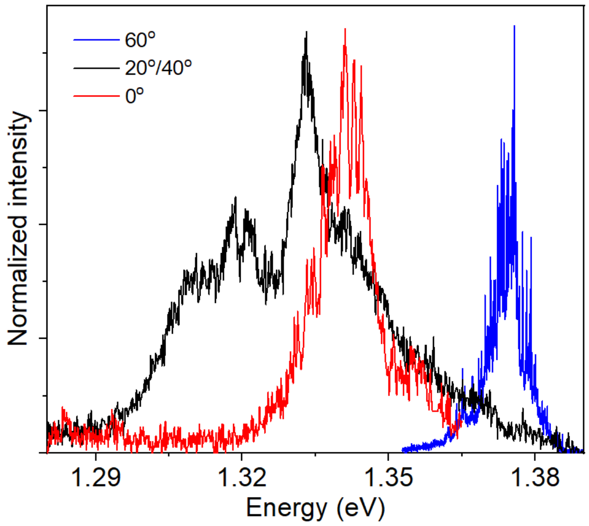

Figure S14 | Twist angle dependence of DAP IX PL emission. Three samples with twist angles 0°, 20(40)°, and 60° are compared. No pronounced sharp peaks were observed for the 20(40)° sample. The PL spectra are normalized to the maximum intensity for each spectrum.

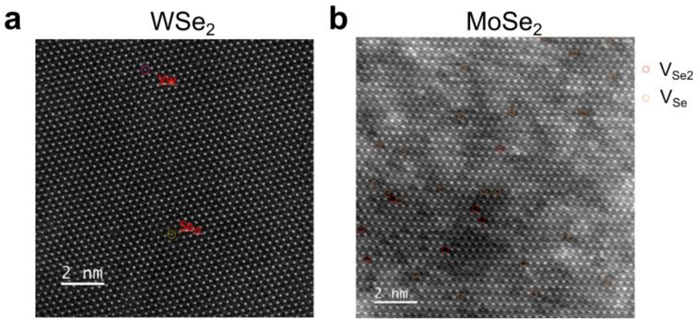

c

Defect type	Density ($\times 10^{12}$ cm^{-2})
V_{Se} in $MoSe_2$	15
$V_{Se}V_{Se}$ in $MoSe_2$	5
V_W in WSe_2	0.6
Se_W in WSe_2	0.6

Figure S15 | Defect characterization using TEM. TEM measurement of **a,** WSe_2 and **b,** $MoSe_2$. The impurities/vacancies are labelled. **c,** Summary of defect type and defect density.

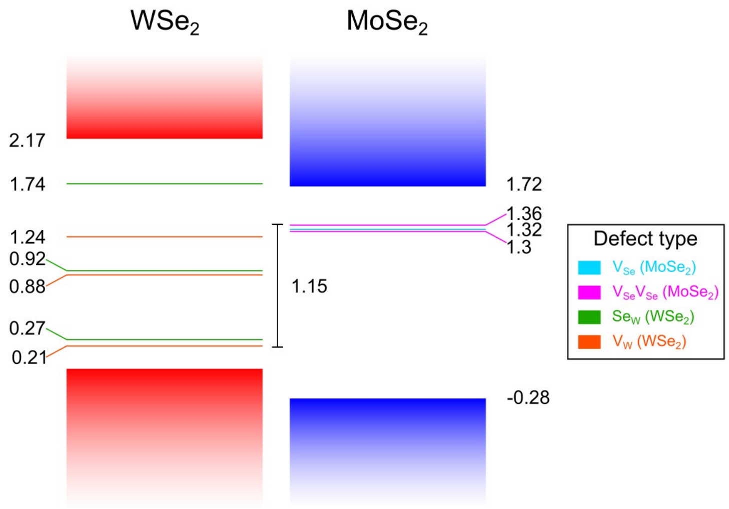

Figure S16 | Donor and acceptor energy levels. The number shown is the distance of the individual level from the WSe_2 valence band maximum (VBM).

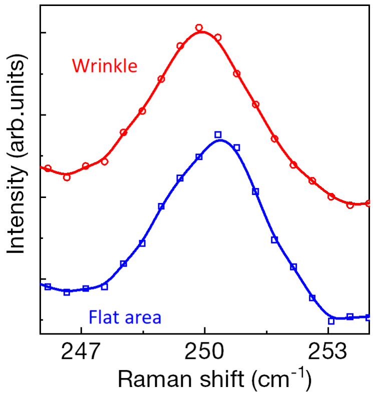

Figure S17 | Comparison between Raman spectrum in wrinkle and flat area. The symbols are measurement results, and the lines are guides for the eye. The WSe_2 A'+E' phonon mode in the wrinkle area is redshifted compared to that in the flat area, indicating a larger strain.

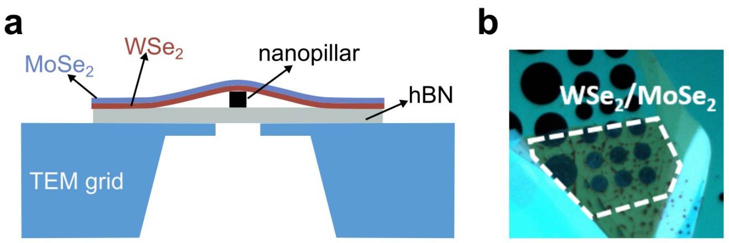

Figure S18 | Heterostructure on pillar sample used in TEM measurement. a, Sample illustration. A standard TEM grid with a 200 nm low strain SiN membrane of a 100×100 μm^2 window with through-holes with diameters ranging from 4 to 20 μm created by FIB was used. The hBN support layer has a thickness of ~10 nm. A PMMA

(A2) layer was spin-coated, and high-dose EBL was used to produce negative nanopillars with amorphous carbon. **b,** Sample image.

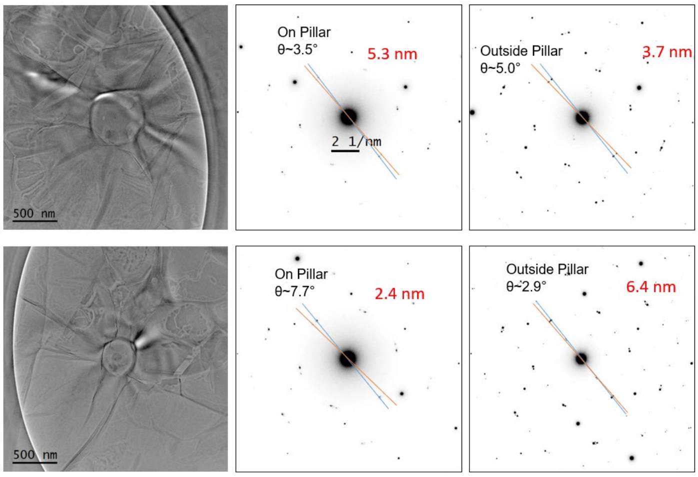

Figure S19 | Effect of nanopillar-induced strain on moiré superlattice. Upper and lower images are images taken from areas around two different nanopillars. The twist angles and moiré superlattice constant (written in red) are shown. The strain on the nanopillar changes the moiré superlattice.

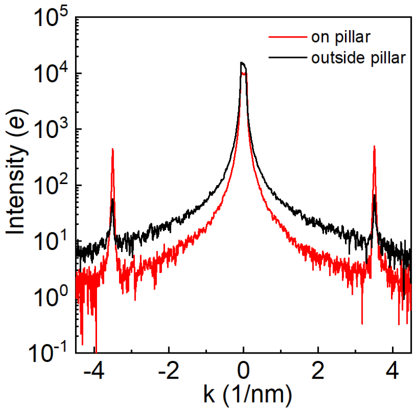

Figure S20 | Linecut of SAED image of WSe_2 on nanopillar sample. The monolayer lattice constant does not change much between pillar and outside pillar areas.

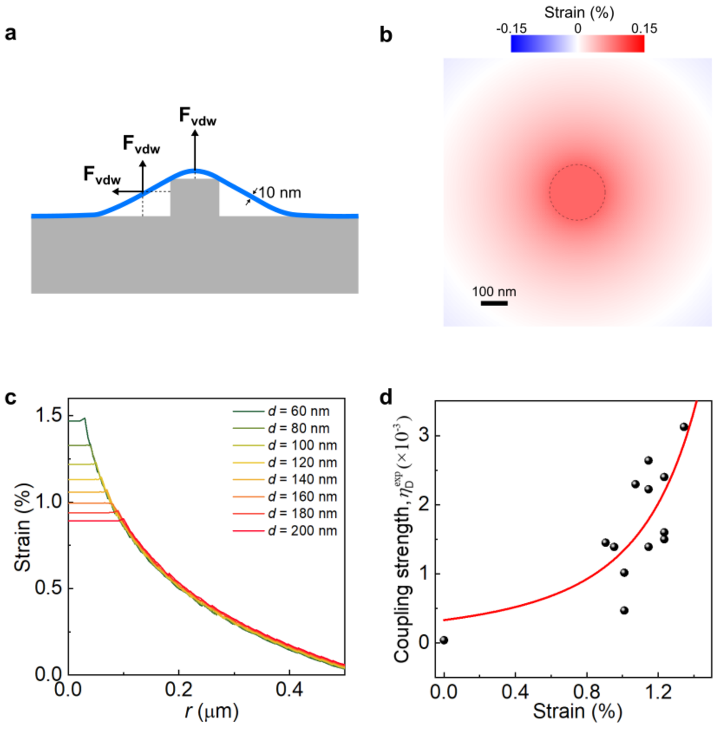

Figure S21 | Strain dependence of coupling strength. a, Strain simulation setting. The van der Waals force direction acting on the 10 nm thick membrane is normal to the nanopillar substrate. **b,** Two-dimensional simulated strain profile**.** The trace of the strain tensor is plotted. The dashed line indicates the position of the edge of the nanopillar with a diameter of 200 nm. **c,** One-dimensional simulated strain profile for various pillars. Here, r is the distance from the centre of the pillar. The strain on the nanopillar area is relatively uniform, with higher strain for smaller pillars. **d,** Coupling strength vs strain on nanopillar. The coupling strength between the band-edge and DAP IX increases with increasing strain. The symbols are measurement results, and the line is the Lorentzian fitting.

**Excitation location**	**Diameter (nm)**	**Strain (%)**	**PL count (kcps)**	**Coupling strength, η_D^{exp} (cps.nm^{-2})**
Dot 1	180	0.95	45	1.39
Dot 2	200	0.91	58	1.45
Dot 3	140	1.07	45	2.30
Dot 4	100	1.23	15	1.50
Dot 5	100	1.23	16	1.60
Dot 6	120	1.15	20	1.39
Dot 7	160	1.01	26	1.02
Dot 8	120	1.15	38	2.64
Dot 9	160	1.01	12	0.47
Dot 10	100	1.23	24	2.40
Dot 11	120	1.15	32	2.22
Dot 12	80	1.34	20	3.13
Flat area	500 (beam diameter)	0.00	10	$\geq$ 0.04

Table S1 | The PL behaviour of DAP IXs on nanopillars array with different diameters.

References

1 Laturia, A., Van de Put, M. L. & Vandenberghe, W. G. Dielectric properties of hexagonal boron nitride and transition metal dichalcogenides: from monolayer to bulk. *npj 2D Mater. Appl.* **2**, 6 (2018).

2 Li, W., Lu, X., Dubey, S., Devenica, L. & Srivastava, A. Dipolar interactions between localized interlayer excitons in van der Waals heterostructures. *Nat. Mater.* **19**, 624-629 (2020).

3 Mahdikhanysarvejahany, F. *et al.* Localized interlayer excitons in $MoSe_2$–WSe_2 heterostructures without a moiré potential. *Nat. Commun.* **13**, 5354 (2022).

4 Shabani, S. *et al.* Deep moiré potentials in twisted transition metal dichalcogenide bilayers. *Nat. Phys.* **17**, 720-725 (2021).

5 Palummo, M., Bernardi, M. & Grossman, J. C. Exciton radiative lifetimes in two-dimensional transition metal dichalcogenides. *Nano Lett.* **15**, 2794-2800 (2015).

6 Aghajanian, M., Mostofi, A. A. & Lischner, J. Tuning electronic properties of transition-metal dichalcogenides via defect charge. *Sci. Rep.* **8**, 13611 (2018).

7 Karni, O. *et al.* Structure of the moiré exciton captured by imaging its electron and hole. *Nature* **603**, 247-252 (2022).

8 Landsberg, P. T., Adams, M. J. & Fröhlich, H. Theory of donor-acceptor radiative and Auger recombination in simple semiconductors. *Proc. R. Soc. London, Ser. A* **334**, 523-539 (1973).

9 Li, E. *et al.* Lattice reconstruction induced multiple ultra-flat bands in twisted bilayer WSe_2. *Nat. Commun.* **12**, 5601 (2021).

10 Zheng, Y. J. *et al.* Point defects and localized excitons in 2D WSe_2. *ACS Nano* **13**, 6050-6059 (2019).

11 Akkoush, A., Litman, Y. & Rossi, M. A hybrid-DFT study of intrinsic point defects in MX_2 (M=Mo, W; X=S, Se) monolayers. *Phys. Status Solidi A* **n/a** (2023).

12 Torun, E., Miranda, H. P. C., Molina-Sánchez, A. & Wirtz, L. Interlayer and intralayer excitons in MoS_2/WS_2 and $MoSe_2/WSe_2$ heterobilayers. *Phys. Rev. B* **97**, 245427 (2018).

13 Dadgar, A. M. *et al.* Strain engineering and Raman spectroscopy of monolayer transition metal dichalcogenides. *Chem. Mater.* **30**, 5148-5155 (2018).

14 Li, H. *et al.* Optoelectronic crystal of artificial atoms in strain-textured molybdenum disulphide. *Nat. Commun.* **6**, 7381 (2015).

15 Rokni, H. & Lu, W. Direct measurements of interfacial adhesion in 2D materials and van der Waals heterostructures in ambient air. *Nat. Commun.* **11**, 5607 (2020).

16 He, Y. *et al.* Strain-induced electronic structure changes in stacked van der Waals heterostructures. *Nano Lett.* **16**, 3314-3320 (2016).

17 Linhart, L. *et al.* Localized intervalley defect excitons as single-photon emitters in WSe_2. *Phys. Rev. Lett.* **123**, 146401 (2019).
